# Imposed work of breathing during high-frequency oscillatory ventilation: a bench study

**DOI:** 10.1186/cc3988

**Published:** 2006-02-01

**Authors:** Marc van Heerde, Huib R van Genderingen, Tom Leenhoven, Karel Roubik, Frans B Plötz, Dick G Markhorst

**Affiliations:** 1 Fellow of Pediatric Intensive Care, Pediatric Intensive Care, Department of Pediatric Intensive Care, VU University Medical Center, Amsterdam, The Netherlands; 2 Medical Physicist, Department of Physics and Medical Technology, VU University Medical Center, Amsterdam, The Netherlands; 3 Biomedical Engineer, Department of Pediatric Intensive Care, Wilhelmina Children's Hospital/University Medical Center, Utrecht, The Netherlands; 4 Biomedical Engineer, Faculty of Biomedical Engineering, Czech Technical University, Prague, Czech Republic; 5 Pediatric Intensivist, Department of Pediatric Intensive Care, VU University Medical Center, Amsterdam, The Netherlands

## Abstract

**Introduction:**

The ventilator and the endotracheal tube impose additional workload in mechanically ventilated patients breathing spontaneously. The total work of breathing (WOB) includes elastic and resistive work. In a bench test we assessed the imposed WOB using 3100 A/3100 B SensorMedics high-frequency oscillatory ventilators.

**Methods:**

A computer-controlled piston-driven test lung was used to simulate a spontaneously breathing patient. The test lung was connected to a high-frequency oscillatory ventilation (HFOV) ventilator by an endotracheal tube. The inspiratory and expiratory airway flows and pressures at various places were sampled. The spontaneous breath rate and volume, tube size and ventilator settings were simulated as representative of the newborn to adult range. The fresh gas flow rate was set at a low and a high level. The imposed WOB was calculated using the Campbell diagram.

**Results:**

In the simulations for newborns (assumed body weight 3.5 kg) and infants (assumed body weight 10 kg) the imposed WOB (mean ± standard deviation) was 0.22 ± 0.07 and 0.87 ± 0.25 J/l, respectively. Comparison of the imposed WOB in low and high fresh gas flow rate measurements yielded values of 1.63 ± 0.32 and 0.96 ± 0.24 J/l (*P *= 0.01) in small children (assumed body weight 25 kg), of 1.81 ± 0.30 and 1.10 ± 0.27 J/l (*P *< 0.001) in large children (assumed body weight 40 kg), and of 1.95 ± 0.31 and 1.12 ± 0.34 J/l (*P *< 0.01) in adults (assumed body weight 70 kg). High peak inspiratory flow and low fresh gas flow rate significantly increased the imposed WOB. Mean airway pressure in the breathing circuit decreased dramatically during spontaneous breathing, most markedly at the low fresh gas flow rate. This led to ventilator shut-off when the inspiratory flow exceeded the fresh gas flow.

**Conclusion:**

Spontaneous breathing during HFOV resulted in considerable imposed WOB in pediatric and adult simulations, explaining the discomfort seen in those patients breathing spontaneously during HFOV. The level of imposed WOB was lower in the newborn and infant simulations, explaining why these patients tolerate spontaneous breathing during HFOV well. A high fresh gas flow rate reduced the imposed WOB. These findings suggest the need for a demand flow system based on patient need allowing spontaneous breathing during HFOV.

## Introduction

Maintenance of spontaneous breathing in mechanically ventilated patients augments ventilation perfusion matching and cardiopulmonary function, reduces sedative requirement and shortens the intensive care stay [1-6]. High-frequency oscillatory ventilation (HFOV) is a useful ventilatory mode for neonatal application [7,8] and it is gaining interest in both pediatric and adult intensive care [9-12]. Neonatal and small pediatric patients can easily breathe spontaneously during HFOV. Muscular paralysis is avoided and only mild sedation needs to be applied to tolerate ventilation and reduce stress. In larger children and adults, however, spontaneous breathing during HFOV is usually not well tolerated because of patient discomfort. The sedation level often has to be high and even muscular paralysis may be necessary [13]. We speculate that this discomfort is caused by a high imposed work of breathing (WOB). The imposed WOB is the work added to the physiologic WOB when patients breathe through a breathing apparatus. This includes work to overcome resistance added by the endotracheal tube, the breathing circuit and the humidification device, and work required to trigger the ventilator demand flow system. A physiologic WOB of 0.3–0.6 J/l is considered normal in a healthy adult [14]. Depending on the ventilator settings, the imposed WOB can contribute as much as 80% to the total work of breathing [15]. The imposed WOB is greatest during continuous positive airway pressure, where the patient performs all the effort required to ventilate [16]. HFOV may in this respect be regarded as super-continuous positive airway pressure.

In a physical sense, work is performed when a transmural pressure (*P*_TM_) changes the volume (*V*) of a distensible structure: *W *= ∫*P*_TM_·d*V*, most often expressed as Joules per liter (J/l). Applied to a breathing apparatus, the imposed WOB is calculated by integrating the pressure measured at the tracheal end of the endotracheal tube (*P*_ETT_) times the volume change: imposed WOB = ∫*P*_ETT_·d*V*. As inspiration is active and expiration is usually passive, only the inspiratory imposed WOB is generally considered. In a SensorMedics HFOV ventilator (3100 A or 3100 B; SensorMedics, Yorba Linda, CA, USA), the imposed WOB is directly related to the breathing-related difference between the set mean airway pressure (MAP) and the *P*_ETT_; the greater the difference, the greater the imposed WOB and thus patient effort. MAP is regulated by a continuous fresh gas flow rate and an expiratory balloon valve. During inspiration of a patient, air is inhaled from the ventilator and the *P*_ETT _level drops. The magnitude of this drop is influenced by the fresh gas flow rate, the endotracheal tube size and the inspiratory flow rate [14].

In order to find a solution to better tolerate spontaneous breathing during HFOV in large pediatric and adult patients we performed a bench test, in which the inspiratory imposed WOB and pressure fluctuations in MAP were assessed for newborn to adult simulations. We evaluated which factors contributed to the imposed WOB in the SensorMedics HFOV ventilator: fresh gas flow rate, endotracheal tube size or inspiratory flow rate.

## Materials and methods

### Bench test set-up

**Figure 1:**
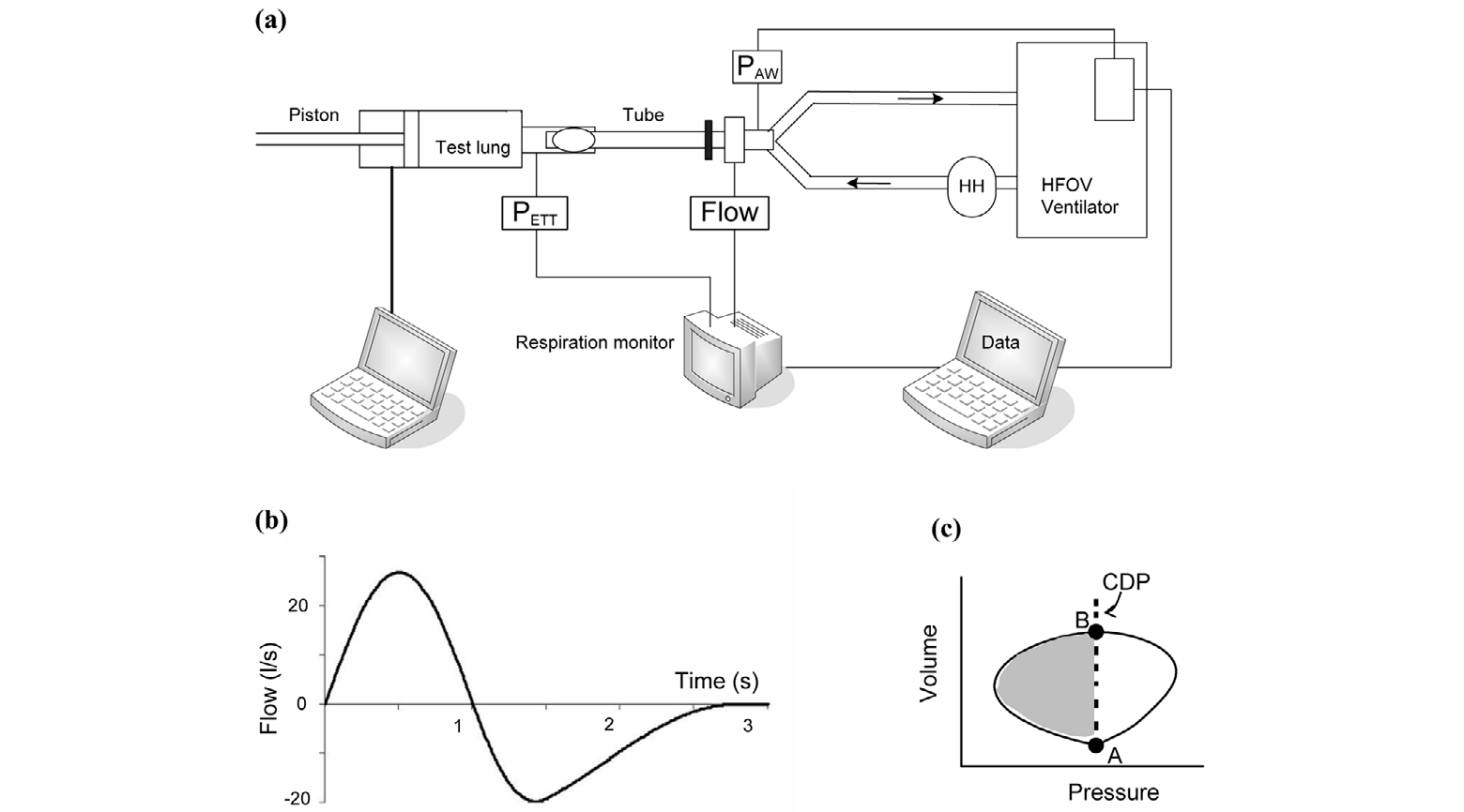
Schematic drawing of the experimental set-up. **(a) **The ventilator circuit of a Sensor Medics 3100 A/B oscillator is connected to a piston-driven test lung by an endotracheal tube. *P*_ETT _and *P*_AW_, pressures in the test lung and in the ventilator circuit, respectively. Flow is measured at the proximal end of the endotracheal tube. HH, heated humidifier; HFOV, high-frequency oscillatory ventilation. **(b) **Simulated spontaneous breath. **(c) **Modified Campbell diagram, where A is the start of inspiration and B is the end of inspiration. The grey area represents the imposed inspiratory work of breathing, calculated using the modified Campbell diagram. CDP, continuous distending pressure.

A custom-made artificial lung was used to simulate a spontaneously breathing subject with variable age (Figure 1a). This test lung consisted of a tube 10 cm in diameter with a computer-controlled piston. A sinusoid flow simulated inspiration of spontaneous breathing, exponential decelerating flow expiration (Figure 1b). The test lung was connected to a HFOV ventilator (3100 A or 3100 B; SensorMedics) with an endotracheal tube (Rüschelit, Rüsch, Kernen, Germany). Different patient circuits were used for each HFOV ventilator (3100 A or 3100 B; SensorMedics). The same heated humidifier was used for both ventilators (MR225 humidification chamber; Fisher and Paykel, Auckland, New Zealand).

The inspiratory airway flow and the expiratory airway flow in the endotracheal tube were measured with a hot-wire anemometer (Florian; Acutronic Medical Systems AG, Hirzel, Switzerland). The tidal volume (*V*_T_) of spontaneous breathing was calculated by flow integration. The *P*_ETT _value was measured using the Florian respiration monitor. The pressure at the Y-piece in the ventilator circuit was measured using the unfiltered electronic signal of the internal pressure sensor of the HFOV ventilator. Flow and airway pressures were sampled at 100 Hz and were stored on a laptop computer for off-line analysis.

HFOV was set to a specific patient size as prescribed by the operator's manuals for management of acute respiratory distress syndrome [17,18]. We tested five patient weight ranges, from newborn to adult (Table 1). The ventilator fresh gas flow rate was set at two different levels: low and high. For all different patient sizes, three *V*_T _levels of normal spontaneous breathing were simulated. The peak inspiratory flow rates that were generated with these *V*_T _levels are also presented in Table 1. Three different sizes of endotracheal tubes were used for each patient size (Table 1). In total, 90 different settings were tested.

### Imposed work of breathing

For each experimental condition, 12–20 breaths were recorded. The inspiratory imposed WOB was calculated for each simulated spontaneous breath, based on the modified Campbell diagram (Figure 1c) [19,20]:

Imposed WOB = Σ_INSP _(CDP - MAP_ETT_)·Δ*V *    (1)

where CDP is the continuous distending pressure or set MAP level on the SensorMedics oscillator, and MAP_ETT _is the mean airway pressure in the test lung. This was calculated by low-pass filtering (Butterworth filter with a cutoff frequency of 10 Hz) of the *P*_ETT _signal to eliminate pressure changes on account of oscillations. The imposed WOB was averaged over all breaths (expressed as J/l).

### Airway pressure

Swings of the pressure in the ventilator circuit due to oscillations were removed by low-pass filtering. As a result, all changes in airway pressure were attributable to the settings chosen to mimic spontaneous ventilation. Pressure fluctuations due to spontaneous breathing (ΔMAP) are expressed as the deviation from CDP (in cmH_2_O). ΔMAP_INSP _is the maximum deviation from the mean airway pressure during inspiration, and ΔMAP_EXP _is the maximum deviation from the mean airway pressure during expiration. ΔMAP_INSP _andΔMAP_EXP _were calculated separately as the inspiratory and expiratory flow patterns of spontaneous breathing differed.

### Statistical analysis

Data are expressed as the mean ± standard deviation. Comparison of means for normally distributed data was performed with an independent *t *test. *P *< 0.05 was considered statistically significant. Linear regression was performed to explore relations between the imposed WOB, the endotracheal tube size, the fresh gas flow rate and the peak inspiratory flow. Statistical analyses were performed using SPSS 11.5 for Windows (SPSS Inc., Chicago, IL, USA).

## Results

### Imposed work of breathing

**Figure 2:**
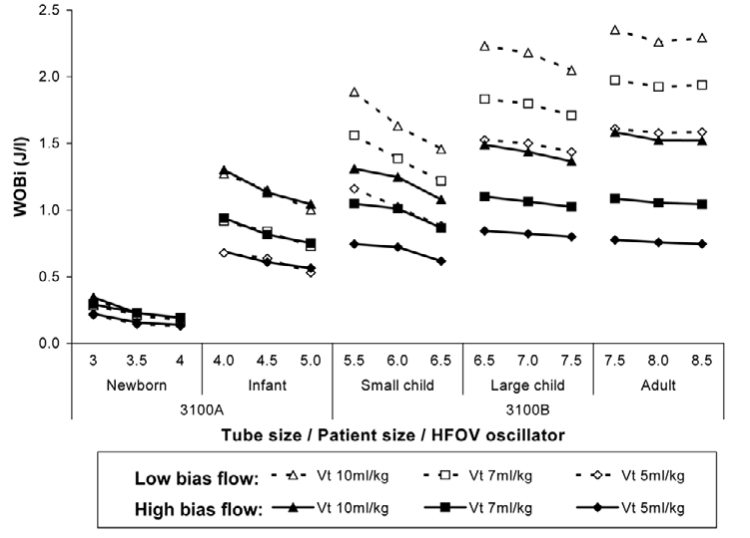
Imposed work of breathing (WOBi) for all simulations. Results for all measurements with the 3100 A and 3100 B SensorMedics oscillators. HFOV, high-frequency oscillatory ventilation; V_T_, tidal volume.

The imposed WOB was 0.22 ± 0.07 J/l for all measurements in the newborn (assumed body weight 3.5 kg) simulations and was 0.87 ± 0.25 J/l in the infant (assumed body weight 10 kg) simulations (Figure 2). Linear regression showed that a high or a low fresh gas flow rate did not independently influence the imposed WOB in these measurements (*P *= 0.64 for newborns and *P *= 0.94 for infants) (3100 A oscillator; SensorMedics). An independent contributor to the imposed WOB was the peak inspiratory flow; a higher peak inspiratory flow increased the imposed WOB (*P *< 0.001). The tube size did not independently contribute to the imposed WOB (*P *= 0.92 for newborns and *P *= 0.92 for infants).

The imposed WOB for the larger pediatric and adult patient size simulations (3100 B oscillator; SensorMedics) was significantly higher in the low fresh gas flow rate condition in comparison with the high fresh gas flow rate condition. The results for the imposed WOB for low flow versus high flow were 1.63 ± 0.32 versus 0.96 ± 0.24 J/l (*P *= 0.01) in the small child (assumed body weight 25 kg) simulation, were 1.81 ± 0.30 versus 1.10 ± 0.27 J/l (*P *< 0.001) in the large child (assumed body weight 40 kg) simulation, and were 1.95 ± 0.31 versus 1.12 ± 0.34 J/l (*P *< 0.001) in the adult (assumed body weight 70 kg) simulation. Independent contributors to the imposed WOB were the fresh gas flow rate (*P *< 0.001) and the peak inspiratory flow (*P *< 0.001). A high fresh gas flow rate decreased the imposed WOB, and a high peak inspiratory flow increased the imposed WOB. The tube size did not independently contribute to the imposed WOB (*P *= 0.07).

### Airway pressure

**Figure 3:**
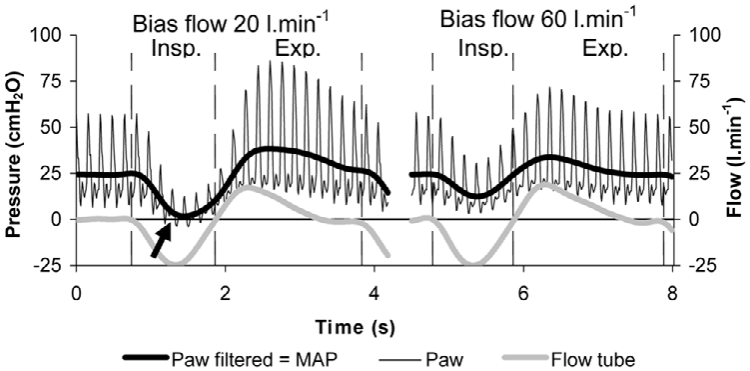
Example of fluctuations in pressure in the ventilator circuit for both low and high fresh gas flow rates. *P*_AW_, unfiltered airway pressure; *P*_AW _filtered, mean airway pressure (MAP) calculated by low-pass filtering of the *P*_AW _signal. Example of the changes in *P*_AW _and *P*_AW _filtered during the simulation for a large child (assumed body weight 40 kg, tidal volume 280 ml), for both low and high fresh gas flow rates. Note that pressure changes decrease with a higher fresh gas flow rate and thus the imposed work of breathing decreases. In this example at the low fresh gas flow rate, the pressure in the ventilator circuit becomes negative as the inspiratory flow exceeds the fresh gas flow rate (arrow). Insp, inspiration; Exp, expiration.

The MAP in the ventilator circuit decreased dramatically during spontaneous breathing, most markedly at a low fresh gas flow rate (Figure 3). In this example the MAP in the ventilator circuit even becomes negative. This effect was observed when the fresh gas flow rate was low and with a *V*_T _of 7 or 10 ml/kg for the large child and adult patient simulations. In these simulations the peak inspiratory flow exceeded the fresh gas flow rate. This triggered the automatic ventilator shut-off, a safety feature of the SensorMedics oscillator.

ΔMAP_INSP _and ΔMAP_EXP _for all measurements in the newborn (assumed body weight 3.5 kg) simulations were not significantly different comparing low and high fresh gas flow rates (Table 2). In the infant (assumed body weight 10 kg) simulations the ΔMAP_INSP _value was significantly lower in the high fresh gas flow rate condition in comparison with the low fresh gas flow rate testing (*P *= 0.002). There was no difference in ΔMAP_EXP _measurements (3100 A; SensorMedics). For pediatric and adult simulations (3100 B oscillator; SensorMedics) the ΔMAP_INSP _and ΔMAP_EXP _values were significantly lower in the high fresh gas flow rate condition in comparison with the low fresh gas flow rate condition (Table 2).

## Discussion

The main result of this study is that the imposed WOB can be markedly increased during HFOV in pediatric and adult patients, especially at low fresh gas flow rates. This can be a good explanation for the discomfort seen in patients breathing spontaneously during HFOV. The fresh gas flow rate and peak inspiratory flow are both strongly related to the imposed WOB. The MAP is not maintained in the breathing circuit when inspiratory flow exceeds the fresh gas flow rate, and this can even lead to ventilator shutdown.

### Work of breathing

Compared with the WOB of a healthy adult (0.3–0.6 J/l), the imposed WOB is high if spontaneous breathing is simulated during HFOV [14]. As the physiologic WOB is not considered in this bench test, the total WOB is even higher in a patient breathing spontaneous during HFOV. An elevated WOB level results in dyspnea and discomfort [21,22]. The optimal workload for critically ill patients is unclear. Research focuses mainly on WOB in the weaning phase [23,24]. A WOB level in the physiologic range (approximately 0.5 J/l in adults) seems to correspond with an optimal workload. Full unloading (for instance, reducing the WOB to zero) induces loss of respiratory muscles. Excessive respiratory muscle loading may cause muscle fatigue and weaning failure [25]. The workload of 0.5 J/l seems to occur not only optimal during weaning, but also in the acute phase of respiratory failure [26,27].

In the pediatric and adult simulations, the imposed WOB exceeded the normal physiologic WOB by as much as 200%. There are very few studies reporting normal WOB values for pediatric patients. WOB in healthy children and adolescents (6–18 years) ranges between 0.1 and 0.6 J/l [28]. In healthy preterm and full-term infants the WOB range is 0.02–0.2 J/l[29]. The optimal WOB during mechanical ventilation for these patients is even more unclear.

Our results show that the level of imposed WOB is high during spontaneous breathing in HFOV. A high fresh gas flow rate in simulations for pediatric and adult patients reduces the imposed WOB, but not within the physiologic range of WOB. It seems logical to aim at a level of imposed WOB in the physiologic WOB range. However, there are no data to support this. An effective way to reduce imposed WOB in HFOV is desirable. Although we did not simulate this condition, a possible solution is to set the fresh gas flow rate to a higher rate. Another solution is the use of a demand flow system instead of the continuous fresh gas flow rate. In order to reduce the imposed WOB, the fresh gas flow rate has to far exceed the peak inspiratory flow. A reasonable suggestion would be the possibility to generate peak fresh gas flow rates comparable with conventional ventilation (approximately 140 l/min) depending on patient need.

Since the imposed WOB does not reflect the isovolumetric breathing effort, the pressure time product per breath was also calculated (data not included). As expected, results for the pressure time product per breath and the imposed WOB were identical. This is explained by the lung model we used for spontaneous breathing, in which the inspiratory and expiratory flows were programmed. The volume changes were imposed, so isovolumetric contraction did not occur.

For the simulations for newborns and infants, the imposed WOB was not influenced by the fresh gas flow rate. This may be explained by the chosen small difference in levels of low and high fresh gas flow rate. Simulations for newborns show a low level of imposed WOB. This is in agreement with the fact that these patients tolerate spontaneous breathing during HFOV. Various factors define the imposed WOB. The endotracheal tube, the breathing circuit, the humidification device and the trigger settings impose the workload. Endotracheal tubes have the greatest effect on flow-resistive work [23]. This seems in contrast with our results, and is explained by the small differences in tube sizes used in our experiments, relative to the large variations in peak inspiratory flow.

### Airway pressure

Large fluctuations in the MAP in the breathing circuit are responsible for a high imposed WOB. They may also lead to unwanted alarms of the ventilator during HFOV, or even to shutdown. Upper and lower alarm limits are routinely set 3–5 cmH_2_O above and below the desired MAP [17,18]. This is a safety precaution against unnoticed MAP changes due to changes in respiratory system compliance, which may lead to alveolar derecruitment or overdistension. Airway pressure fluctuations exceeded the alarm limit of 5 cmH_2_O in all simulations on the SensorMedics 3100 B ventilator. In the simulations for smaller patient size, alarm limits of 3 cmH_2_O above and below the CDP were sufficient to avoid alarms – although in most other measurements the alarm limits had to be set wide to stop alarms, interfering with patient safety in a clinical setting.

If the peak inspiratory flow exceeded the fresh gas flow rate, this led to ventilator shutdown. This triggered the automatic ventilator shut-off, a safety feature of the SensorMedics oscillator.

## Limitations of the study

The imposed WOB is strongly related to the choice of *V*_T_, respiratory rate, breathing pattern and tube size. In this *in vitro *study we aimed to choose realistic test conditions. However, *in vivo *conditions may differ from our bench test. In the lung model used only the imposed WOB can be evaluated. Patient or total work cannot be assessed. During HFOV the lungs can expand to high levels of end expiratory lung volume near the total lung capacity. At high levels of end expiratory lung volume the work of breathing will increase as a result of an increase in elastic work. The *V*_T _levels used for simulations were fixed. The *V*_T _level that a patient can generate is influenced by the level of end expiratory lung volume. Only shallow breathing is possible at levels of end expiratory lung volume near the total lung capacity. In this lung model we are not able to evaluate these effects on patient WOB and on total WOB. These findings need validation in clinical practice.

## Conclusion

The imposed WOB is considerable in spontaneous breathing in pediatric and adult patients during HFOV and is a good explanation for observed patient discomfort. A high fresh gas flow rate decreased the imposed WOB, but not sufficiently. Large swings in airway pressure complicate the setting of safe alarm limits and can even lead to ventilator malfunction. In clinical practice it is reasonable to consider the level of the fresh gas flow rate. In order to minimize the imposed WOB and to allow at least shallow spontaneous breathing during HFOV, the fresh gas flow rate has to be set at a maximum level.

## Key messages

• 	In a bench test, simulated spontaneous breathing during HFOV results in a high level of the imposed WOB.

• 	The high level of imposed WOB explains the discomfort seen in patients breathing spontaneously during HFOV.

• 	A high fresh gas flow rate reduces the imposed WOB, but not to an acceptable level.

• 	Fluctuation of the MAP, on account of spontaneous breathing, interferes with safety settings.

• 	A possible solution is the use of a demand flow system instead of continuous fresh gas flow.

## Abbreviations

CDP = continuous distending pressure; HFOV = high-frequency oscillatory ventilation; MAP = mean airway pressure; ΔMAP_EXP _= maximum deviation from the mean airway pressure during expiration; ΔMAP_INSP _= maximum deviation from the mean airway pressure during inspiration; *P*_ETT _= pressure at end tracheal tube; *P*_TM _= transmural pressure; *V*_T _= tidal volume; WOB = work of breathing.

## Competing interests

The authors declare that they have no competing interests.

## Authors' contributions

MvH designed the study, conducted the bench study, analyzed the results and drafted the manuscript. HRvG, TL and KR assisted in designing the study and participated in interpreting the results. FBP participated in interpreting the results and drafting the manuscript. DGM discovered the phenomenon under clinical conditions, assisted in designing the study, and participated in interpreting the results and drafting the manuscript. All authors read and approved the final manuscript.

## Figures and Tables

**Table 1:** Spontaneous breathing simulation and ventilator settings

	Newborn	Infant	Small child	Large child	Adult
Assumed weight (kg)	3.5	10	25	40	70
Spontaneous breathing simulation					
Respiratory rate (/min)	35	30	25	20	12
Tidal volume (ml/kg)/peak inspiratory flow (l/min)	5/2.6	5/6.3	5/13	5/17	5/18
	7/3.6	7/8.9	7/19	7/24	7/25
	10/5.1	10/12	10/27	10/34	10/36
Inspiratory/expiratory ratio	1:2				
Ventilator settings					
HFOV ventilator	3100 A	3100 A	3100 B	3100 B	3100 B
Tube inner diameter (mm)	3.0, 3.5, 4.0	4.0, 4.5, 5.0	5.5, 6.0, 6.5	6.5, 7.0, 7.5	7.5, 8.0, 8.5
Fresh gas flow rate (l/min)	15/20	20/40	20/60	20/60	20/60
Continuous distending pressure (cmH_2_O)	18	25	25	25	25
Proximal pressure amplitude (cmH_2_O)	35	50	50	50	50
Oscillation frequency (Hz)	10	8	8	6	6

HFOV ventilator, SensorMedics 3100 A or SensorMedics 3100 B high-frequency oscillatory ventilation ventilator.

**Table 2:** Maximum deviation of the mean airway pressure from the set continuous distending pressure during inspiration and expiration

	ΔMAP_INSP _(cmH_2_O)			ΔMAP_EXP _(cmH_2_O)		
	
	Low fresh gas flow rate^a^	High fresh gas flow rate^b^	*P *value	Low fresh gas flow rate^a^	High fresh gas flow rate^b^	*P *value
SensorMedics 3100 A ventilator						
Newborn	2.17 ± 0.24	1.75 ± 1.04	Not significant	0.83 ± 0.27	0.71 ± 0.44	Not significant
Infant	6.85 ± 1.34	4.56 ± 1.24	0.002	3.73 ± 1.77	3.59 ± 1.70	Not significant
SensorMedics 3100 B ventilator						
Small child	17.0 ± 3.11	8.91 ± 2.11	< 0.001	8.90 ± 2.49	5.21 ± 1.70	0.002
Large child	23.0 ± 2.60	12.8 ± 2.62	< 0.001	13.2 ± 2.01	8.76 ± 1.66	< 0.001
Adult	25.5 ± 2.51	13.7 ± 3.50	< 0.001	15.9 ± 2.97	8.34 ± 2.18	< 0.001

ΔMAP_INSP_, maximum deviation of mean airway pressure from continuous distending pressure during inspiration; ΔMAP_EXP_, maximum deviation of mean airway pressure from continuous distending pressure during expiration. ^a^Low fresh gas flow rate: 3100 A ventilator, 15 ml/min and 3100 B ventilator, 20 ml/min. ^b^High fresh gas flow rate: 3100 A ventilator, 20 ml/min and 3100 B ventilator, 60 ml/min.
